# Clinical Outcomes of Patients with Oral Cavity Squamous Cell Carcinoma and Retropharyngeal Lymph Node Metastasis Identified by FDG PET/CT

**DOI:** 10.1371/journal.pone.0079766

**Published:** 2013-11-11

**Authors:** Jing-Ren Tseng, Tsung-Ying Ho, Chien-Yu Lin, Li-Yu Lee, Hung-Ming Wang, Chun-Ta Liao, Tzu-Chen Yen

**Affiliations:** 1 Department of Nuclear Medicine and Molecular Imaging Center, Chang Gung Memorial Hospital at Linkou, Taoyuan, Taiwan, R.O.C.; 2 Department of Otorhinolaryngology, Head and Neck Surgery, Chang Gung Memorial Hospital at Linkou, Taoyuan, Taiwan, R.O.C.; 3 Department of Radiation Oncology, Chang Gung Memorial Hospital at Linkou, Taoyuan, Taiwan, R.O.C.; 4 Department of Pathology, Chang Gung Memorial Hospital at Linkou, Taoyuan, Taiwan, R.O.C.; 5 Department of Medical Oncology, Chang Gung Memorial Hospital at Linkou, Taoyuan, Taiwan, R.O.C.; 6 Department of Head and Neck Oncology Group, Chang Gung Memorial Hospital at Linkou, Taoyuan, Taiwan, R.O.C.; Stanford University Medical Center, United States of America

## Abstract

**Purpose:**

Retropharyngeal lymph node (RPLN) metastasis is an uncommon finding in patients with oral cavity squamous carcinoma (OSCC). We sought to investigate the clinical outcomes, clinicopathological characteristics, and the priority of treatment with curative intent in OSCC patients with RPLN involvement.

**Methods and Materials:**

Between January 2007 and January 2011, we identified 36 patients with primary RPLN metastases (n = 10) or RPLN relapse (n = 26). The follow-up continued until June 2013. Disease-specific survival (DSS), disease-free survival (DFS), and the potential benefits of salvage therapy served as the main outcome measures.

**Results:**

The 2-year DSS and DFS rates of untreated patients with RPLN involvement were 20% and 24%, respectively. Level IV/V neck lymph node involvement was an adverse prognostic factor for DSS (*P* = 0.048) and DFS (*P* = 0.018). All of the patients presenting with neck lymph node involvement at level IV/V died within 6 months. Among patients who were treated for RPLN relapse, the 2-year DSS and DFS rates from the relapse day were 12.8% and 9.6%, respectively. Concomitant contralateral neck lymph node metastases (N2c) were associated with lower 2-year DSS (*P* = 0.005) and DFS (*P* = 0.011) rates. Moreover, five (55%) of the nine patients with recurrent disease in the contralateral RPLN had distant metastases within 6 months. Salvage therapy yielded the maximum survival benefit in patients without N2c disease and ipsilateral RPLN involvement alone (*P* = 0.005).

**Conclusion:**

OSCC patients with RPLN involvement have poor outcomes. The risk factor for definitive treatment in OSCC patients with FDG PET/CT defined RPLN disease in primary disease was neck lymph node involvement at level IV/V and N2c and/or contralateral RPLN disease in recurrent disease. Treatment efforts with curative intent should be tailored according to individual risk factors.

## Introduction

The presence of retropharyngeal lymph node (RPLN) metastasis is an uncommon finding in patients with oral cavity squamous cell carcinoma (OSCC) [1]. To date, only a handful of case reports have documented RPLN metastases in patients with OSCC [2]–[4]. In limited series of patients with non-nasopharyngeal squamous cell carcinoma of the head and neck, the involvement of RPLN has been associated with poor outcomes [5], [6]. There are several reasons why patients with RPLN involvement can have a poor prognosis, including the aggressive nature of the disease, the difficult surgical access to the retropharyngeal space, and time delays in the diagnosis [1]–[6].

Because RPLN is clinically impalpable, the detection of RPLN is based on imaging modalities such as CT and MRI. Unfortunately, the accuracy of the current imaging modalities remains suboptimal. Morrisey et al. have reported that the sensitivity and specificity of CT for RPLN detection are 50% and 70%, respectively [7]. MRI is superior to CT [8], [9], and it has been traditionally considered the preferred modality until the introduction of FDG PET/CT, which has a better diagnostic utility in identifying RPLN metastasis in head and neck cancers [10].

Unusual site neck recurrence in OSCC (including the prelaryngeal area, the parotid area, and the retropharyngeal area) indicates the presence of an aggressive disease, which in turn results in a dismal prognosis. In our historical cohort, the overall survival at 5 years in patients with unusual site neck recurrence was 4%, compared with 45% of pN+ patients [1]. However, the clinical impact of RPLN in OSCC patients has not been characterized in detail. We therefore designed the current study to investigate the clinical outcomes, clinicopathological characteristics, and the priority of treatment with curative intent in this group of patients.

## Materials and Methods

This study was designed as a retrospective analysis of prospectively collected data. Since this study involved retrospective review of existing data, approval from the Institutional Review Board of the Chang Gung Memorial Hospital (CGMH) at Linkou (Number: 101-1556B) was obtained, but without specific informed consent from patients. The Institutional Review Board of Chang Gung Memorial Hospital specifically waived the need for consent for this observational study. The study protocol was approved by the local Medical Ethics Committee with compliance to the guidelines of the Declaration of Helsinki. The written informed consent regarding detail information publication (as outlined in PLOS consent form) was obtained from individual in this manuscript. All of the data were securely protected (by delinking identifying information from the main data sets), made available only to investigators, and analyzed anonymously.

### Patients

Between January 2007 and January 2011, 2678 OSCC patients were admitted to Chang Gung Memorial Hospital to undergo FDG PET/CT scanning for staging and/or restaging. We identified by PET/CT a total of 38 OSCC patients with RPLN involvement. All of the patients had positive findings in both PET/CT and CT/MRI. However, two patients were excluded from the analysis because curative-intent treatment was not feasible due to the presence of concomitant distant metastases diagnosed in the pretreatment phase. Therefore, a total of 36 patients were included in the study. All of the study participants underwent extensive evaluations, including physical examinations, blood/chemistry tests, chest radiography, bone scans, liver ultrasonography, FDG PET/CT, and CT or MRI scans of the head and neck. Patient staging was performed according to the criteria of the 2002 American Joint Committee on Cancer (AJCC), 6^th^ Edition.

### Treatment

The primary tumors were excised with ≥1 cm margin, and tumor margin tissue was subsequently cryosectioned. If a margin was positive, additional tissue was excised to ensure that the margin was free of tumors. Under microscopic pathological examination, clear margins were defined as greater than 5 mm in both the peripheral and deep margins. If patients had clinically positive neck nodes, classical radical or modified neck dissections (level I–V) were used, whereas supraomohyoid neck dissections (level I–III) were performed in patients with clinically negative neck nodes. The RPLN was not routinely dissected. Surgical defects were immediately repaired with either primary closure or reconstructed by plastic surgeons using free or local flaps.

Patients with risk factors such as pT4 tumors, positive metastatic lymph nodes, or close margins (≤4 mm) received postoperative radiotherapy (RT). Postoperative RT was prescribed as 2 Gy per fraction per day, given 5 days per week within 4 to 8 weeks after surgery. Patients received irradiations via intensity-modulated radiation therapy techniques. The prophylactic RT dose was 46–50 Gy for comprehensively encompassing all of the lymphatics at risk (including the regional lymphatic basin) with 1–2 cm margins. An additional boost of 60 Gy was applied to the primary site and the involved nodal area with 0.5–1 cm margin. Concurrent chemoradiation (CCRT) was performed in patients with microscopic positive margins, more than two positive metastatic nodes (pN2b) and/or nodal extracapsular spread, with a total RT dose of 66 Gy. CCRT was administered with cisplatin 30 mg/m^2^ weekly or cisplatin 50 mg/m^2^ biweekly plus daily 800 mg of tegafur and 60 mg of leucovorin [11].

In patients who were not amenable to surgical excision of the primary tumor or RPLN, the RT dose to the gross lesions was escalated to 72 Gy. In patients with recurrent disease, the re-irradiation dose was reduced to 60–66 Gy to avoid severe morbidity. Palliative chemotherapy was given to patients in poor general conditions.

### FDG PET/CT imaging protocol and interpretation

All of the FDG PET/CT scans were performed on a Discovery ST 16 PET/CT scanner (GE Healthcare, Milwaukee, WI, USA). Patients fasted for 6 hours before scanning, and images were acquired 50 min after the intravenous injection of 370 to 444 MBq (10 to 12 mCi) ^18^F-FDG. All of the patients underwent head to mid-thigh scans. CT data were used for both attenuation correction and fusion with attenuation-corrected PET images. Images were reconstructed using ordered subsets expectation maximisation with four iterations and ten subsets. All images (including PET, CT, and PET/CT) were displayed in axial, coronal, and sagittal views; PET data were also displayed in a rotating maximum-intensity projection.

To interpret the abnormal foci on FDG PET/CT, we used our previously described scoring criteria for RPLN [12]. The degree of FDG uptake with respect to the background vascular activity (ranging from similar to markedly higher) was scored on a 5-point scale, as follows: 0, no abnormal uptake; 1, benign; 2, probably benign; 3, probably malignant; and 4, definitely malignant. Scores of 3–4 were considered positive, whereas scores of 0–2 were considered negative. In CT or MRI images, any visible node in the median retropharyngeal group and those of the lateral group with the shortest axis ≥5 mm were considered malignant, as were signs of central necrosis or extracapsular spread [13].

### Data Analysis

The follow-up was continued until June 2013 or until the patients' death. The time intervals were calculated from the first day of RPLN identification to the event of interest for primary RPLN and from primary treatment to the event for recurred RPLN. The disease-specific survival (DSS) rate was defined as the survival until death from cancer-related causes. The disease-free survival (DFS) rate was defined as the interval until loco/regional relapse or distant metastases. The events for the determination of DFS were defined by pathological evidence, imaging findings, or physical examination. The survival curves were calculated using the Kaplan-Meier method and tested for significance using the log-rank test. All calculations were performed using the SPSS software, version 16.0 (SPSS Inc., Chicago, IL, USA). Two-tailed *P* values <0.05 were considered statistically significant.

## Results

### Clinicopathological Characteristics of OSCC patients with RPLN metastasis

Table 1 shows the clinical characteristics of the 36 OSCC patients with RPLN involvement. Ten patients were diagnosed during staging whereas the remaining 26 cases were identified during restaging of recurrent disease. The median follow-up time was 14 months (mean, 17.4±12.9 months; range, 2–51 months). At the end of follow-up, 31 (86.1%) patients expired, 3 (8.3%) patients were alive with disease, and 2 (5.6%) patients were alive without disease.

**Table 1:** General characteristics of patients with retropharyngeal lymph node involvement (n = 36).

Characteristic, n (%)	Primary RPLN (n = 10)	Recurrent RPLN (n = 26)
Male sex	10 (100)	24 (92)
Age, years	53.9±13.40	50.27±10.52
Betel quid chewing	7 (70)	17 (65)
Cigarette smoking	9 (90)	16 (61)
Alcohol drinking	8 (80)	16 (61)
LocationAlveolar ridgeHard palateMouth floorBuccal mucosaRetromolar trigoneTongue	4 (40)1 (10)0 (0)4 (40)0 (0)1 (10)	2 (8)1 (4)2 (8)7 (27)7 (27)7 (27)
Stage* #Stage IStage IIStage IIIStage IVAStage IVB	0 (0)0 (0)0 (0)4 (40)6 (60)	6 (23)8 (31)3 (11)7 (27)2 (8)
Treatment modality#SurgerySurgery + CCRTSurgery + RTCCRTChemotherapy	2 (20)3 (30)0 (0)4 (40)1 (10)	14 (54)9 (35)3 (11)0 (0)0 (0)
OutcomeLocal relapseRegional lymph node relapseDistal metastasesMortality	1 (10)2 (20)3 (30)9 (90)	10 (38)17 (65)8 (27)22 (85)

RPLN, retropharyngeal lymph node; CCRT, concurrent chemoradiation; RT, radiotherapy.

* Stage based on clinical classification if patient did not undergo surgery.

# Indicates the initial stage and the treatment modality in patients with recurrent RPLN.

Thirty-one (86.1%) of the 36 patients received operations, whereas the remaining five patients (13.9%) underwent either definitive CCRT or chemotherapy alone. Of the 5 patients, 4 were inoperable (surgically unresectable disease in three and medically inoperable condition in one) and one refused surgery. Among the 31 operated patients, 15 (48.4%) underwent adjuvant CCRT/RT therapy. Fifteen patients (48.4%) had pStage IV disease, and 12 (38.7%) were diagnosed with pT4 tumors. Fifteen (48.4%) patients had pN+ disease, and six (19.4%) showed extracapsular spread (ECS). All of the five operated patients who had primary RPLN occurrence had pStage IV disease, pN+ at level I–III, and ECS+.

All of the study participants had at least two FDG PET/CT scans, of which one was performed before the treatment and the other after the definite treatment. The mean SUVmax of the main tumor and the RPLN SUV nodal-max were 13.82±5.40 and 8.92±3.96, respectively. The RPLNs were identified contralaterally of the main tumor site in 11 (31%) patients, whereas 25 (69%) were on the same side.

### Clinical course of OSCC patients with primary RPLN metastasis

**Figure 1:**
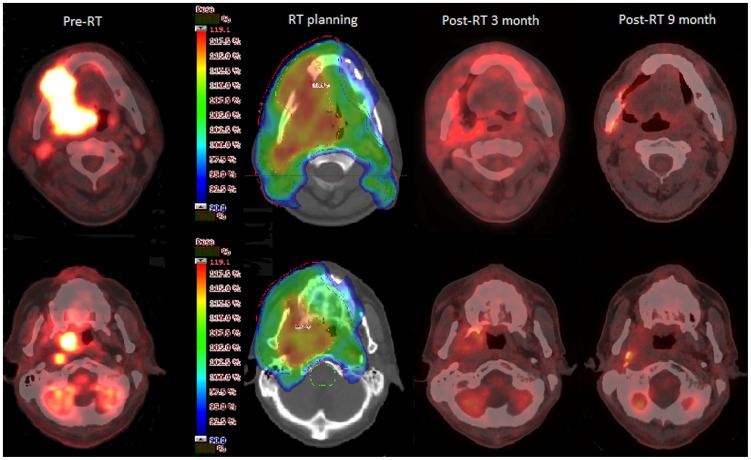
A 35-year-old male patient was diagnosed with right-sided lower gum cancer, stage cT4bN2bN0. The patient underwent curative-intent treatment with concurrent chemo-radiotherapy using simultaneous integrated boost intensity-modulated radiotherapy (SIB-IMRT) with dose-painting (dose escalation within a gross target volume). The images show the sequential changes in the main tumor (upper panel) as well as in the ipsilateral RPLN (lower panel). The patient had no evidence of recurrence after 51 months of follow-up.

**Figure 2:**
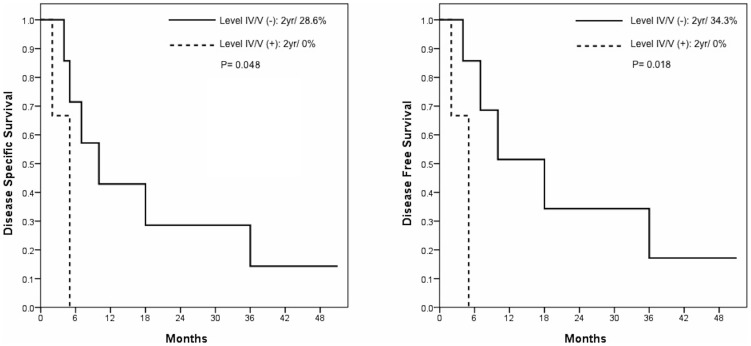
Kaplan-Meier plots of DSS and DFS in primary OSCC patients with retropharyngeal lymph node metastasis identified by FDG PET/CT.

Ten patients were identified as having RPLN involvement at the initial presentation (Table 2). The 2-year DSS and DFS rates were 20% and 24%, respectively. Seventy percent of patients died within 12 months, and only one patient was alive at 51 months (Fig 1). Survival analysis showed that level IV/V neck lymph node involvement was a marginally significant risk factor for DSS (*P* = 0.048) and a significant adverse prognostic factor for DFS (*P* = 0.018) (Fig 2). Three patients with a diagnosis of first primary OSCC and level IV/V neck lymph node involvement died within 6 months, whereas the remaining 7 patients had a median survival time of 10 months (mean, 18.7±18.0 months; range, 4–51 months). During the follow-up period, three patients developed distant metastases (one had bone metastasis at 9 months; one had lung metastasis at 15 months; and the remaining one had bone metastasis at 23 months after the documentation of RPLN involvement).

**Table 2:** Clinical characteristics of patients with primary retropharyngeal lymph node involvement (n = 10).

No.	Sex	Age	Site	Stage	Treatmentmodality	RPLN site	RPLN SUVnodal-max	Interval between RPLN identification and clinical events (months)				Salvage	Endpoint (months)/Outcome
								RPLN recurrence	Neck recurrence	Tumor recurrence	Distant metastasis (sites)		
1	M	35	Gum	cT4bN2b	CCRT	Ipsilateral	9.5	-	-	-	-	-	51/NER
2†	M	62	Gum	pT4aN2b	S+CCRT	Ipsilateral	10.68	-	23	-	23 (bone)	-, palliation (C/T)	36/DOD
3	M	60	Buccal	cT4bN2c	CCRT	Ipsilateral	5.1	-	15	15	15 (lung)	-, palliation (C/T)	18/DOD
4	M	49	Hard palate	cT4bN2c	C/T	Ipsilateral	12.00	-	-	-	-	-	7/DOD
5*	M	53	Tongue	pT4aN2c	S	Ipsilateral	6.8	-	-	-	-	-	5/DOD
6*	M	48	Buccal	cT4bN2b	CCRT	Ipsilateral	10.00	-	-	-	-	-	2/DOD
7	M	53	Gum	pT2N2b	S	Contralateral	4.3	-	-	-	-	-	5/DOD
8*	M	43	Gum	pT4bN2b	S+CCRT	Ipsilateral	4.24	-	-	-	-	-	5/DOD
9	M	51	Buccal	pT4aN2c	S+CCRT	Ipsilateral	7.4	-	-	-	9 (bone)	-	10/DOD
10	M	85	Buccal	cT4bN2c	CCRT	Contralateral	3.6	-	-	-	-	-	4/DOD

M, male, F, female, S, surgery; RPLN, retropharyngeal lymph node; CCRT, concurrent chemoradiation; C/T, chemotherapy; SUV, standardized uptake value; NER, no evidence of recurrence; DOD, died of cancer or related disease.

† The patient had two malignancies (i.e., lung small cell carcinoma and oral cavity squamous cell carcinoma).

* The patient had level IV/V neck lymph node involvement.

### Clinical course of OSCC patients with recurrent RPLN metastasis and the role of salvage therapy

**Figure 3:**
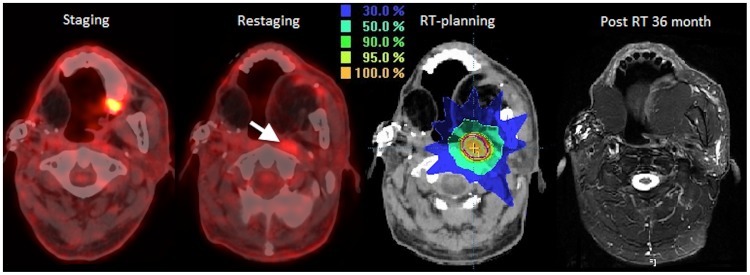
A 55-year-old male patient was diagnosed with second primary left retromolar cancer, stage pT1N1N0. RPLN recurrence was documented at 6 months after primary treatment. Concurrent chemo-radiotherapy using intensity-modulated radiotherapy (IMRT) was prescribed as salvage therapy. The patient had no evidence of recurrence after 43 months of follow-up.

**Figure 4:**
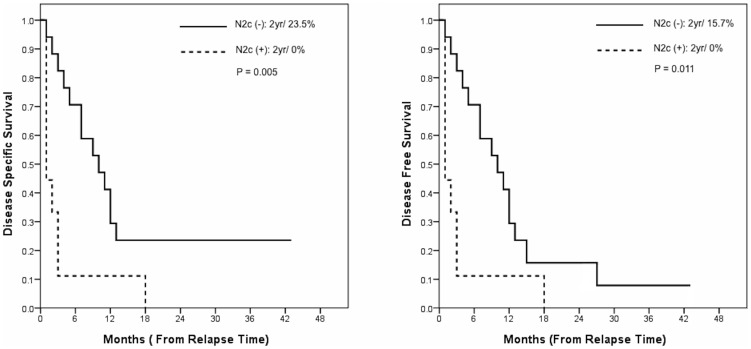
Kaplan-Meier plots of DSS and DFS in OSCC patients who had disease relapse at the retropharyngeal lymph node identified by FDG PET/CT.

In the 26 patients who were diagnosed with RPLN disease at restaging, the 2-year DSS and DFS rates from the day of relapse were 12.8% and 9.6%, respectively. Of this group, 76.9% of patients died within 12 months, and only one patient was alive at 43 months (Fig 3). Survival analysis identified contralateral neck lymph nodes involvement as a significant risk factor for DSS and DFS (*P* = 0.005 and 0.011, respectively) (Fig 4). Compared with patients with ipsilateral cN+ disease (n = 17; median, 10 months; mean, 11.4±11.0 months; range, 1 to 43 months), the probability of dying within 18 months after RPLN identification in subjects with contralateral cN+ disease was 100% (n = 9; median, 1 months; mean, 3.4±5.5 months; range, 1 to 18 months). During the follow-up period, 8 patients developed distant metastases, and five (62.5%) of them had contralateral RPLN involvement. All of the five patients with contralateral RPLN recurrence displayed subsequent distant metastases within 6 months after RPLN identification (one had celiac lymph node metastasis; another had lung and bone metastases; and the others had lung metastases).

Eleven (42.3%) patients received salvage therapy. Of them, ten received CCRT with curative intent and one had surgery plus CCRT. Overall, salvage therapy showed a marginally significant survival benefit in patients with recurrent RPLN (P = 0.048). However, in the subgroup of patients with contralateral neck lymph node involvement, the only patient who received salvage CCRT died within one month after the completion of the treatment course. No significant survival benefit from salvage therapy was observed in the subgroup of patients with isolated ipsilateral neck lymph node involvement (Table 3; P = 0.132). However, this group of patients had a better median survival time compared with subjects who did not receive salvage therapy (12 months versus 7 months). After the exclusion of patients with contralateral RPLN involvement (80% distant metastasis rate within 6 months), salvage therapy yielded its maximum survival benefit in the subgroup of patients with ipsilateral RPLN alone and without contralateral neck lymph node metastases (P = 0.005).

**Table 3:** Clinical characteristics of patients with retropharyngeal lymph node recurrence (n = 26).

No.	Sex	Age	Site	pStage	Treatment mode	RPLN site	RPLN SUVnodal-max	Interval between primary treatment and clinical events (months)				Salvage	Follow-up# (Months)/Outcomes
								RPLN recurrence	Neck recurrence	Tumor recurrence	Distant metastasis (sites)		
Patient with isolated ipsilateral neck lymph node involvement													
1	M	55	Retromolar	pT1N1	S	Ipsilateral	4.30	8	-	-	-	+, CCRT	43/NER
2	M	51	Buccal	pT2N0	S	Ipsilateral	12.00	14	14	-	-	+, CCRT	18/AWD
3	M	68	Hard palate	pT2N0	S	Ipsilatera	8.09	12	-	-	-	+, CCRT	14/AWD
4	M	49	Mouth floor	pT4aN2b	S+CCRT	Contralateral	5.1	4	4	-	4 (celiac lymph node)	-, palliation(C/T)	29/AWD
5	M	47	Retromolar	pT1N0	S	Contralateral	10.65	11	-	-	17 (lung)	+, CCRT	8/DOD
6	M	68	Tongue	pT1N2b	S+CCRT	Contralateral	16.40	4	4	-	-	-, palliation(C/T)	9/DOD
7	M	39	Retromolar	pT4aN1	S+RT	Contralateral	6.9	10	-	10	13 (lung)	+,CCRT	10/DOD
8	M	45	Buccal	pT4aN2b	S+CCRT	Contralateral	5.38	5	5	-	5 (lung, bone)	-, palliation(C/T)	3/DOD
9	M	51	Tongue	pT1N0	S	Ipsilateral	10.25	14	14-	-	-	+, CCRT	25/DOD
10	M	43	Mouth floor	pT2N0	S+RT	Ipsilateral	12.00	8	8	-	-	-, palliation (C/T)	6/DOD
11	F	47	Tongue	pT2N0	S	Ipsilateral	5.1	6	-	-	-	+, CCRT	9/DOD
12	M	50	Retromolar	pT4bN0	S+CCRT	Ipsilateral	6.87	15	-	-	-	-, palliation (C/T)	9/DOD
13	M	50	Buccal	pT1N1	S	Ipsilateral	5.9	26	26	-	-	+, S+CCRT	17/DOD
14	M	38	Buccal	pT2N2b	S+CCRT	Ipsilateral	7.89	12	-	17	-	+, CCRT	15/DOD
15*	M	37	Retromolar	pT2N0	S+CCRT	Ipsilateral	6.76	17	-	17	-	+, CCRT	7/DOD
16	M	51	Retromolar	pT1N0	S	Ipsilateral	6.99	4	4	4	-	-, palliation (C/T)	8/DOD
17	M	53	Gum	pT4aN0	S+RT	Ipsilateral	14.28	3	-	-	3 (lung)	-, palliation(C/T)	3/DOD
Patients with contralateral lymph node involvement													
18	M	52	Tongue	pT1N0	S	Ipsilateral	14.00	10	10	-	10 (lung)	palliation (C/T)	3/DOD
19	M	56	Tongue	pT2N0	S+CCRT	Contralateral	6.33	2	2	-	-	-	18/DOD
20	M	45	Gum	pT4bN2b	S+CCRT	Ipsilateral	18.30	10	10	10	-	-	1/DOD
21	F	31	Tongue	pT2N0	S	Ipsilateral	4.50	1	1	1	-	+,CCRT	3/DOD
22	M	78	Buccal	pT2N0	S	Ipsilateral	13.34	4	4	4	-	palliation (C/T)	1/DOD
23	M	42	Retromolar	pT1N0	S	Contralateral	9.72	4	4	4	-	palliation (C/T)	1/DOD
24	M	55	Buccal	pT1N0	S	Ipsilateral	7.40	8	8	-	8 (lung)	palliation (C/T)	1/DOD
25	M	42	Buccal	pT4aN2c	S+CCRT	Contralateral	18.10	1	1	1	1 (lung)	palliation (C/T)	1/DOD
26	M	64	Tongue	pT2N1	S	Contralateral	9.10	5	5	5	-	palliation (C/T)	2/DOD

M, male, F, female, S, surgery; RPLN, retropharyngeal lymph node; CCRT, concurrent chemoradiation; RT, radiotherapy; C/T, chemotherapy; DOD, died of cancer or related disease; SUV, standard uptake value; NER, no evidence of recurrence; AWD, alive with disease.

* The patient had an unusual involvement of the parotid gland.

# Follow-up was calculated from the first day of RPLN recurrence identification.

## Discussion

The involvement of RPLN is extremely rare in OSCC patients, especially in those with primary disease. The prognosis of patients with RPLN involvement is generally dismal. Our group has conducted a continuous cohort study with FDG PET/CT in OSCC since January 2007. We hypothesized that the use of FDG PET/CT-based staging and restaging protocols would lead to the identification of a higher number of OSCC patients with RPLN disease at an earlier stage, which would in turn improve the survival rates. We therefore retrospectively retrieved our data to investigate the clinical outcomes, clinicopathological characteristics, and priority of treatment with curative intent in OSCC patients with RPLN involvement.

In this study, only 10 of the 2,678 OSCC patients (0.37%) included in the data bank of our cancer center had RPLN involvement at the initial presentation. In a previous report, the authors identified two subjects with RPLN involvement from a cohort of 326 OSCC patients (0.61%) [4]. Two distinct hypotheses have been offered to explain the occurrence of RPLN in OSCC patients. One hypothesis involves the reverse lymphatic flow from the upper internal jugular lymph nodes to the RPLN. The second hypothesis postulates a role of the direct lymphatic drain from the oral mucosa to the RPLN in posterior invading oral cancer [3]. In patients treated with surgery, the coexistence of risk factors − including advanced tumor status (100% [5/5] in primary disease and 26.9% [7/26] in recurrent disease), neck lymph node metastasis (100% [5/5] in the primary group and 38.5% [10/26] in the relapse group), level I–III involvement (100% [5/5] in the primary group and 34.6% [9/26] in the relapse group), and extracapsular spread (100% [5/5] in the primary group and 3.8% [1/26] in the relapse group) − indicated that RPLN involvement was more common in highly aggressive tumors.

The presence of neck node metastases to level IV/V identified by PET were associated with poor prognosis in primary disease, whereas contralateral neck lymph node metastasis predicted a poor outcome in patients with recurrent disease. However, these data should be taken with caution because of the low number of subjects with primary (n = 10) and recurrent disease (n = 26). These results were in line with our previous reports showing that level IV/V involvement and contralateral neck lymph nodes metastases are important predictors of outcomes [14]–[16]. PET/CT allowed an early identification of RPLN metastases. A combined evaluation of the involvement of RPLN and other regional nodes by PET/CT (low and contralateral neck lymph node metastases) may be helpful not only to define the priority of treatment with curative intent but also avoid unnecessary curative attempts in patients with poor prognosis.

Most patients with RPLN metastases had been previously treated (72.2%, 26/36). Therefore, the potential usefulness of salvage therapy needs to be discussed. In the subgroup of patients with isolated ipsilateral neck lymph node metastasis, salvage therapy did not result in a significant survival benefit (P = 0.132). Furthermore, four of five (80%) OSCC patients with contralateral RPLN metastases had distant metastasis within 6 months after relapse (Table 3). When patients with contralateral RPLN were excluded, salvage therapy yielded its maximum survival benefit in the subgroup of subjects with ipsilateral RPLN alone and without contralateral neck lymph node metastases (P = 0.005). Despite its lack of prognostic significance in patients with recurrent disease, our findings demonstrate that salvage therapy should not be recommended in OSCC patients with contralateral RPLN metastases.

FDG PET/CT-based imaging protocols in OSCC patients are clinically useful to identify the involvement of RPLN and regional nodes, as well as distant metastases. However, some important questions remain unanswered. For example, the optimal time for detection and the optimal salvage treatment are still unclear. In addition, the potential usefulness of novel treatment strategies and the clinical value of molecular prognostic factors should be evaluated in future well-designed studies.

Some caveats of this study merit comment. This was a single-center retrospective analysis conducted in an endemic betel nut chewing area; therefore, the results may not be generalizable to other countries. In addition, patients were treated with different primary regimens (mainly radical surgery plus adjuvant RT/CCRT vs. CCRT) and the impact of a high incidence of second primary or even third primary tumors remains unclear. However, the current report also has several strengths. To our knowledge, this is the largest case series reported to date. Despite the rarity of this condition, the risk factors that contributed to the occurrence of RPLN were clearly addressed. Finally, we were able to identify several independent prognostic factors, including contralateral RPLN metastases and the involvement of level IV/V or contralateral neck lymph nodes.

In summary, our results demonstrate that the incidence of RPLN metastasis in OSCC patients is less than 1%. More than two thirds of such cases were identified among patients with recurrent disease. Furthermore, our findings indicate that treatment attempts with curative intent should be offered to patients without level IV/V neck node metastasis at primary staging by PET/CT. Such an approach may result in improved clinical outcomes. Because of the high morbidity and the low survival rates in recurrent OSCC patients with RPLN disease, salvage therapy should be targeted to patients with ipsilateral RPLN identified by PET/CT in the absence of contralateral RPLN or contralateral neck node disease.
